# Development of the Human Tooth

**Published:** 1893-12

**Authors:** E. P. Beadles


ARTICLE V.

### DEVELOPMENT OF THE HUMAN TOOTH.

BY DR. E. P. BEADLES.

Read before the Virginia State Dental Association, August 8, 1893.

The histology of the teeth is too voluminous a subject for full treatment in a short paper, as this must necessarily be; hence, I have selected a small portion, viz.: The periods of calcification, the formation of dentine, and the formation of enamel. These will be treated on the surface merely. A few salient points will be brought out, which we can readily carry in our minds.

There is a wide field for prophylactic treatment in connection with the development of the teeth of the human race, which the future will demand of the dental profession to enter. It is well known that each succeeding generation brings with it more inferior teeth than was known in the preceding. We have in our chairs now children younger than ever before. Now, there must be a cause for this, and this cause it developes upon us to discover. Having found it, as an honest profession we will endeavor to remove it.

If I understand the object of these papers before your association, they are intended to put briefly and simply what the books tell us in detail about the subjects we discuss.

Dentine, as is universally admitted, is an offspring of connective tissue produced by the papilla, which is a formation of embryonal tissue crowded with medullary capsules. It begins to appear about the end of the second and beginning of the third month of intra-uterine life, at a time when the extremity of the epithilial cord has begun to flatten and assume a cup-shape. The cavity of this cup is filled with the papilla which sends prolongations along the outer wall of the cup, the future sack of the tooth.

At the beginning of the fifth month the odontoblasts are noticed. These are not direct dentine formers, but are provisional formations from which arise medullary corpuscles, and these are changed into the basis-substance of the dentine. In a word, we see then that the dentine from the first is a formation of connective tissue, first visible in the shape of a knob-like protuberance termed the papilla. Second, the papilla is composed of medullary (or marrow-like, or tumor-like) tissue, holding an irregular myxomatus net-work, originally scanty, but later on freely supplied with arteries, veins and capillaries Third, shortly before the formation of dentine (in the fifth month of fœtal life) there appear at the periphery of the papilla elongated corpuscles, known as odontoblasts. From the odontoblasts are sent off the offshoots, which are dentine fibers. Fourth, the medullary corpuscles are transformed into basis-substance, which is the seat of the deposit of lime-salts. Next comes the development of the cementum, which I omit, except to say that it develops after birth, when the root and its dentine have been fully formed. At birth the crowns only of the temporary teeth are present, there being no trace of the roots.

The epithelial cord of the enamel organ is a formation of the epiblast. In the same manner as the nerve centers (brain and syinal cord) are products of the epiblast, greatly changing their character in the further course of development, the epithelial cord gives rise to the myxomatus tissue of the enamel organ. The epithelial cord arises from a furrow, lined with epithelium; about the sixth week of intra-uterine life, and grows obliquely downward into the connective tissue, which latter produces the papilla about the third fœtal month.

After the formation of the enamel organ the epithelial cord is dissolved into clusters, which are partly transformed into fibrous connective tissue. The remnants of the external epithelium, as well as those of the epithelial cord, very probably furnish the material for the increase of the enamel after the original enamel organ has been exhausted.

The epithelial cord of the temporary tooth furnishes a lateral offshot for the formation of the permanent tooth. The papilla of the latter appear about the seventh month of intra-uterine life.

I now take up the periods of calcification, which are important.

After the temporary teeth are developed and have served their purpose they are then removed by resorption of their roots, and their places taken by the permanent set. A good deal has been said about the cause of this resorption and many opinions expressed. My own is that they are taken up by the same process that any foreign body is absorbed by the system and discarded. When the permanent tooth comes in contact with the end of the temporary root the nerve is severed and the tooth becomes a foreign body, there being little supply from the periosteum. When the permanent tooth fails to develop these teeth sometimes remain in the mouth for a number of years.

In the microscopical examination of dense animal structure, or such tissues as are made up of lime-salts, it is seen that they, like vegetable structures, have periods of growth and rest, which are illustrated by concentric layers or zonal shades, and that, while these conditions are normal, they are both intensified and modified by the genius presiding over the function of nutrition.

For the temporary teeth, we know that by the seventh week of intra-uterine life, and when the embryo is less than one and one-quarter inches in length, preparation is made for the development of the enamel germ, followed in the ninth week by the dentine germ. In the seventeenth week we find the border line between the enamel and dentine germs receiving depositions of the salts of lime. By the nineteenth week the same process has reached the molars, and from this period until the fortieth week, or time of birth, the growth of the tooth-germs and their calcification progress simultaneously. At birth the calcification of the crowns of the eight incisors is quite complete; the four cuspids and four first molars are fully two thirds calcified; and the four temporary second molars have their crowns for half their length solidified by the same process. At the end of the following three months the infant enters into the critical period of its life, and from a glance at the condition of the twenty deciduous teeth and their progressive developmental change, it is reasonable indeed to assume that the conditions have not a little to do with the various abnormal systemic disturbances to which the child is subject at the time.

As early as the fifteenth week of embryonic life preparation is made for the development of the four first permanent molars, and following close upon these in the sixteenth week is the inflection giving rise to the enamel organ for the twenty anterior teeth, and from this period until birth the germs for twenty-four of the permanent teeth are passing through their several progressive stages preparatory to receiving the salts of lime. At birth, then, the child has not only the deciduous teeth largely advanced toward calcification, but has germs of twenty-four permanent teeth, in twelve of which calcification commences the first year. The germ of the second molar makes its appearance the third month, and that of the third molar the third year after birth.

The permanent teeth during the periods of calcification are very sensitive to morbid changes of the system, and any abnormal condition, even though of short duration, is almost sure to leave its mark upon the crowns of the teeth.

The four first permanent molars and the eight incisors receive during the first year a portion of their lime-salts, and by the end of the third year twenty-four of the thirty-two teeth are in this process of development.

The fifth year the second permanent molars commence calcifying. The fact that the third molars are developed during the period of childhood and youth, when there is such a demand for all substances which go to make up the body, is one reason why these teeth are generally lacking in development; hence, they are often of little value.

It will be seen from the above how important it is for us to carry in our minds these periods of calcification, if we are to do anything in the way of prophylactic treatment.—*Southern Dental Journal.*

